# Magnetic resonance imaging findings among young adults with low back pain at Nsambya hospital

**DOI:** 10.1186/s12880-022-00830-5

**Published:** 2022-06-04

**Authors:** Komakech Richard Lukecha, Erem Geoffrey, Mubuuke A. Gonzaga, Bugeza Sam

**Affiliations:** Department of Radiology, School of Medicine, College of Health Sciences, Makerere University, Kampala, Uganda

**Keywords:** Lumbar spine, Degeneration disc disease, Ligamentum flavum hypertrophy, Marginal osteophyte, Magnetic resonance imaging

## Abstract

**Background:**

Studies on MRI findings among patients with LBP have been conducted; especially among adolescents and young adult population in developed countries. However, MRI lumbar spine evaluation findings in young adult patients with low back pain in Uganda is not known. The purpose of this study was to determine the MRI findings and their correlation to clinical features in young adult patients with low back pain in Nsambya hospital.

**Methods:**

This was a descriptive cross sectional study. One hundred and fifty-seven patients with low back pain in the 18–39 year age group underwent MRI lumbar spine evaluation. The MRI changes in the lumbar spine and correlation to clinical features were determined. Correlation was assessed by Pearson chi square tests (Fisher’s exact test) and *p* values reported at 0.05 level of significance.

**Results:**

Of the 157 patients 129 (82.2%) had severe pain, whereas ninety (57.3%) had pain that had lasted more than 10 weeks. Sixty-five (41.4%) patients were found to have MRI evidence of disc desiccation, majority (61%) of whom had multiple level disease, mostly involving the lowest 2 disc levels. Facet joint arthropathy (47.8%), marginal osteophyte (31.8%) and disc contour irregularity [disc bulge] (31.2%) were other common MRI features seen. There was an association between duration of pain and limb weakness, and development of marginal osteophytes. There was also association between clinical presentation and disc bulge.

**Conclusions:**

The MRI finding of disc degeneration among young adult patients with LBP is higher than reported. Age and pain distribution are predictors of developing disc desiccation.

## Background

Low back pain (LBP) is a common health problem with a global prevalence of 20% [1]. The annual prevalence of LBP in Africa is 57%, whereas in Uganda is 20% [2]. It peaks between age 35 and 55 years [3, 4]. This high number of people having low back pain has made physicians overwhelmed by work. In Africa, Plain x-ray is the only available imaging modality in most primary health care centers [5, 6]. This has provided equivocal results to patients who have undergone lumbar spine evaluation [7], thereby making patients’ treatment frustrating and unrewarding [8].

Magnetic resonance imaging (MRI) has played a significant role in evaluating lumbar-sacral spine as it is able to show clearly any anomaly of the vertebrae, intervertebral disks, spinal cord, the neuroforamina, ligamentum flavum, facet joints and the longitudinal ligaments. The clinicians and most patients now prefer MRI to radiographic evaluations because of its high spatial resolution ability providing images that will offer diagnosis of a disease, monitoring treatment response and follow up of patients since it provides conclusive results. It is also used to determine extent of a disease and in follow up of patients. MRI is indicated in most conditions such as severe progressive neurologic deficit, persistent low back pain with features of radiculopathy, spinal stenosis, or when a patient is to undergo surgery where plain x-ray would provide inconclusive results [9].

Certain lifetime occupation and activities have influenced the development of lumbar spine degeneration diseases [10], such activities include heavy weight lifting or any work that requires over bending of waist [11]. A study conducted by Takatala et al. (2011) among Finnish young adults with low back pain using MRI found that disc degeneration (Modic changes, Schmorl’s nodes), disc bulge, radial tears, spondylosis and sacroiliac joint abnormality were common among sportsmen. High intensity zone lesion was more common among women whereas disc herniation was common among men; disc extrusion was least seen in both sexes. The degenerative disc findings are commonly found at L5–S1 level, whilst high intensity zone lesions are mostly seen at L4–L5 [12].

The Modic changes had no gender difference with Modic type 1 being more common than type 2 and were located adjacent to a disc degeneration [13]. Other studies that gave similar results were conducted in Kuwait, China, USA, Sweden and UK; and found that disc degeneration was the most common MRI finding [13].

The purpose of this study was to determine the MRI findings and their correlation to clinical features in young adult patients with low back pain in Nsambya hospital.

## Methods

### Design

This was a cross-sectional descriptive study in which images of young adults with LBP were reviewed.

### Setting

The study was conducted in the department of radiology of Nsambya Hospital. The hospital offers both outpatient and in-patient services. It is a 361-bed capacity private-not-for-profit hospital located in the southern part of Kampala city approximately 3 km from the city center. It has a radiology department equipped with 1.5Tesla Siemens MRI, 128 slides computed tomography (CT) machine, 04 ultrasound machines, conventional x-ray machine and mammography machine. The department on average receives 86 patients with low back pain for MRI evaluation every month and between 15 and 17 will be young adult patients. However, this number increases during national inter school’s/Universities sports seasons.

### Participants and sampling

Consecutive sampling of young adults aged between 18 to 39-years old who are to undergo MRI lumbosacral spine evaluation after referral to the department.

### Data collection

Participants were recruited at the MRI room reception station and written informed consent were obtained. Bio-data, Clinical detail and level of physical activity were obtained and recorded.

The MRI scan of the lumbar spine was performed with a 1.5 Tesla MRI machine (Siemens Medical Systems, model—Espree, Town—Henkestr, County—Erlangen) using a dedicated receive—only spine coil, and a standard protocol specification for young adult in sagittal T1W, T2W, T2W STIR, T2W myelo, sequences. Coronal/axial reformats at levels T12-S1 were obtained. In suspected neoplastic and inflammatory processes the images were acquired in T1WI with Contrast, and gradient echo (GRE) sequences.

The MRI images were viewed at the picture archiving and communication system (PACS) GE (Centricity, GE Medical Systems) workstations. The corresponding radiological reports were made by the principal investigator and supervised by two consultant radiologists with substantial experience in neuroradiology imaging. Any difference in opinion was settled by consensus.

Data was captured for every image using a data collection form that was de-identified to exclude any unique identifier that would reveal the identity of the image. All study data forms were checked for accuracy, completeness and consistency regularly and any identified errors was corrected on the spot. All forms with completed data were sorted, and coded with unique study identifiers.

### Data analysis

Frequency and proportions of variable were determined using descriptive statistics. Continuous variables were summarized in median and inter quartile range. Median and interquartile ranges were used because the data were not normally distributed.

Multiple logistic regression analysis was done using Stata 13.0 to find clinical and MR factors associated with spine image characteristics. Odds ratios (ORs)) were calculated to measure the effect of an independent variable on the outcome variable. Bivariate analysis, for independent variables was used and those which have *p* values less than 0.2 at 95% confidence intervals were considered for multivariate analysis. Interaction and confounding were assessed in the regression model to determine the factors that are associated with low back pain. Variables with *p* < 0.05 was considered statistically significant.

### Ethical issues

Permission to conduct the study was sought from, and granted by the Department of Radiology. Institutional approval for the study was sought from, and granted by the Makerere University School of Medicine Research and Ethics Committee on June 24th, 2019 (REC REF 2019-096). All study activities and procedures were conducted per Good Clinical and Laboratory Practice starting June 27th, 2019 and ended on June 26th, 2020. All patients were briefed about the study; background, aim, risks, benefits and expectations for participation before being consented to participate. Written informed consent (in English or local language) were sought from each of the prospective participants in the study prior to enrolment and data collection. All data and results generated from this study were kept confidential and were only accessed by a few authorized personnel. Preliminary examination of patients to assess whether or not they had red flags, other diseases for which they could not undergo MRI or be part of the study was conducted at the hospital’s orthopedic department. Illegible participants were advised on the next step according to the routine standard practice at the department.

## Results

**Fig. 1:**
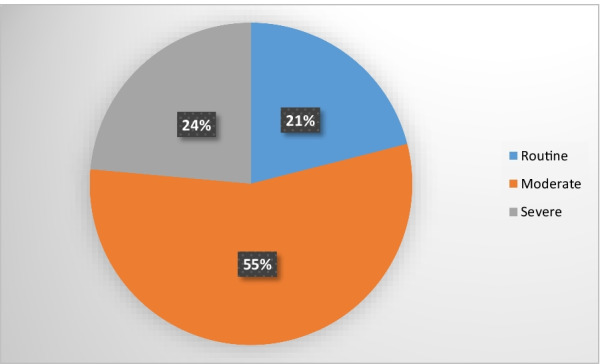
Levels of activity of patients

Of the 157 patients (74 males and 83 females; age range 18 to 39 years, median [IQR] 33 [14–23]) who were evaluated, 138 (88%) [48 males and 90 females] were found to have lumbar spine diseases as shown by Table 1. Majority of patients (79%) had indulged in moderate to intense activities [as showed by Fig. 1].

**Table 1:** Sociodemographic profiles of the patients

Variable	Frequency (n = 157)	Percentage (%)
*Sex*		
Female	83	52.9
Male	74	47.1
*Age, median (IQR)*	33 (29–38)	
18–22	13	08.2
23–27	24	15.3
28–32	40	25.5
33–37	38	24.2
38–42	42	26.8
*Occupation*		
Housewife	12	7.6
Students	24	15.3
Business	39	24.8
Professional (formally employed)	82	52.2

Eighty-seven (55.4%) patients had gradual onset of pain whereas 70 (44.6%) had sudden onset; 129 (82.2%) patients had severe pain and most of which were burning in nature (n = 116, 73.9%). Majority of the patients had radiating pains (n = 97, 61.8%) that was frequently aggravated by bending down (n = 67, 42.7%) and alleviated by lying down (n = 141, 89.8%).

The median duration of pain was 4 weeks, with a minimum duration of 1 week and maximum of 14 weeks. Sixty-seven (42.7%) had acute pain whereas ninety (57.3%) patients had chronic pain. Six (3.8%) patients had lower limb weakness; 4 (66.7%) had weakness of both lower limbs as shown by Table 2.

**Table 2:** Pain characterisation of the patients

Variable	Frequency (n = 157)	Percentage (%)
*Duration of pain*		
≤ 6 weeks	80	50.9
> 6—11 weeks	25	15.9
> 12 weeks	52	33.2
*Onset of pain*		
Gradual	87	55.4
Sudden	70	44.6
*Side of the back affected*		
Left	17	10.8
Right	29	18.5
Both	111	70.7
*Quality of pain*		
Aching	41	26.1
Burning	116	73.9
*Severity of pain*		
Mild	00	00
Moderate	28	17.8
Severe	129	82.2
*Aggravating factors*		
Bending	67	42.7
Sitting	64	40.8
Standing	26	16.5
*Alleviating factors*		
Walking	7	4.5
Sitting	9	5.7
Lying down	141	89.8
*Distribution*		
Localised	60	38.2
Radiating	97	61.8

**Fig. 2:**
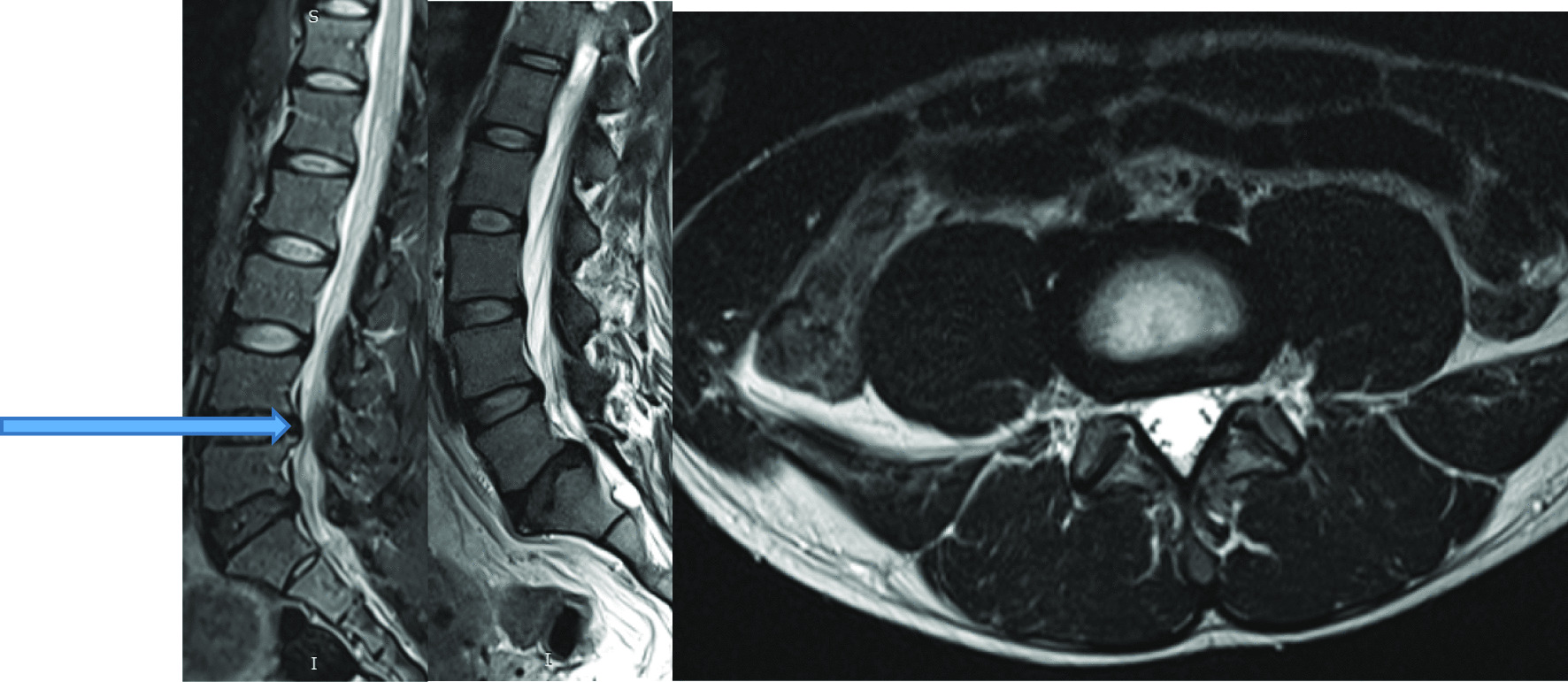
Sagittal and axial planes of T2W sequence showing bilateral broad based disc bulge and spinal canal stenosis (blue arrow) at L4/L5 and L5/S1 in a 28 years old female patient who presented with sciatica for three weeks

**Fig. 3:**
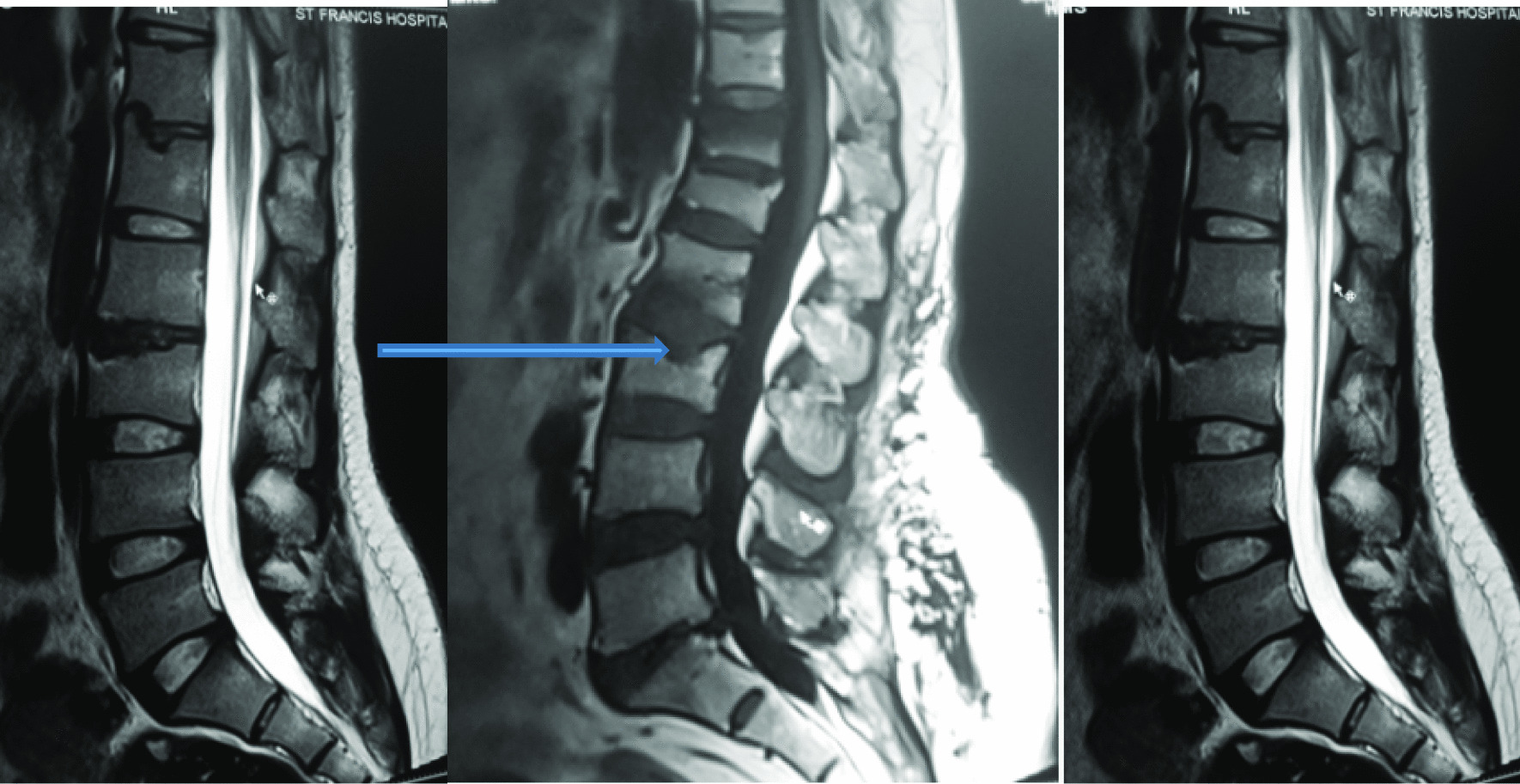
T2W sequence in Sagittal plane showing disc desiccation at L2/L3 and Schmorls node (blue arrow) at vertebrae L1 and L3

**Fig. 4:**
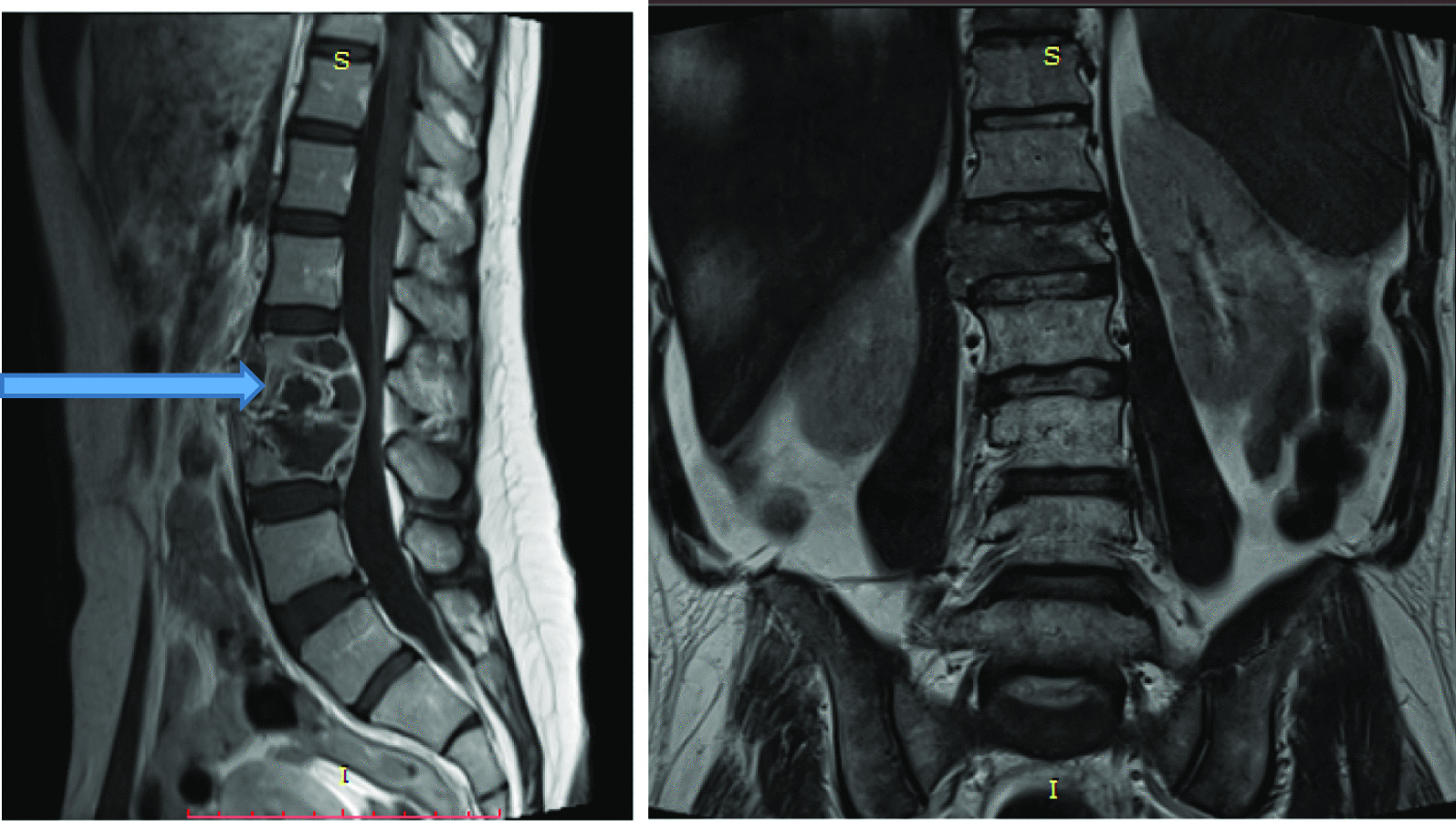
T1W sequence in Sagittal plane and T2W sequence in coronal plane showing multiple irregularly shaped thick rim enhancing lesions involving the bodies of vertebrae L2 and L3, and the intervertebral disc L2/L3 with associated spinal canal stenosis, para-spinal space involvement and longitudinal ligaments disruption

**Fig. 5:**
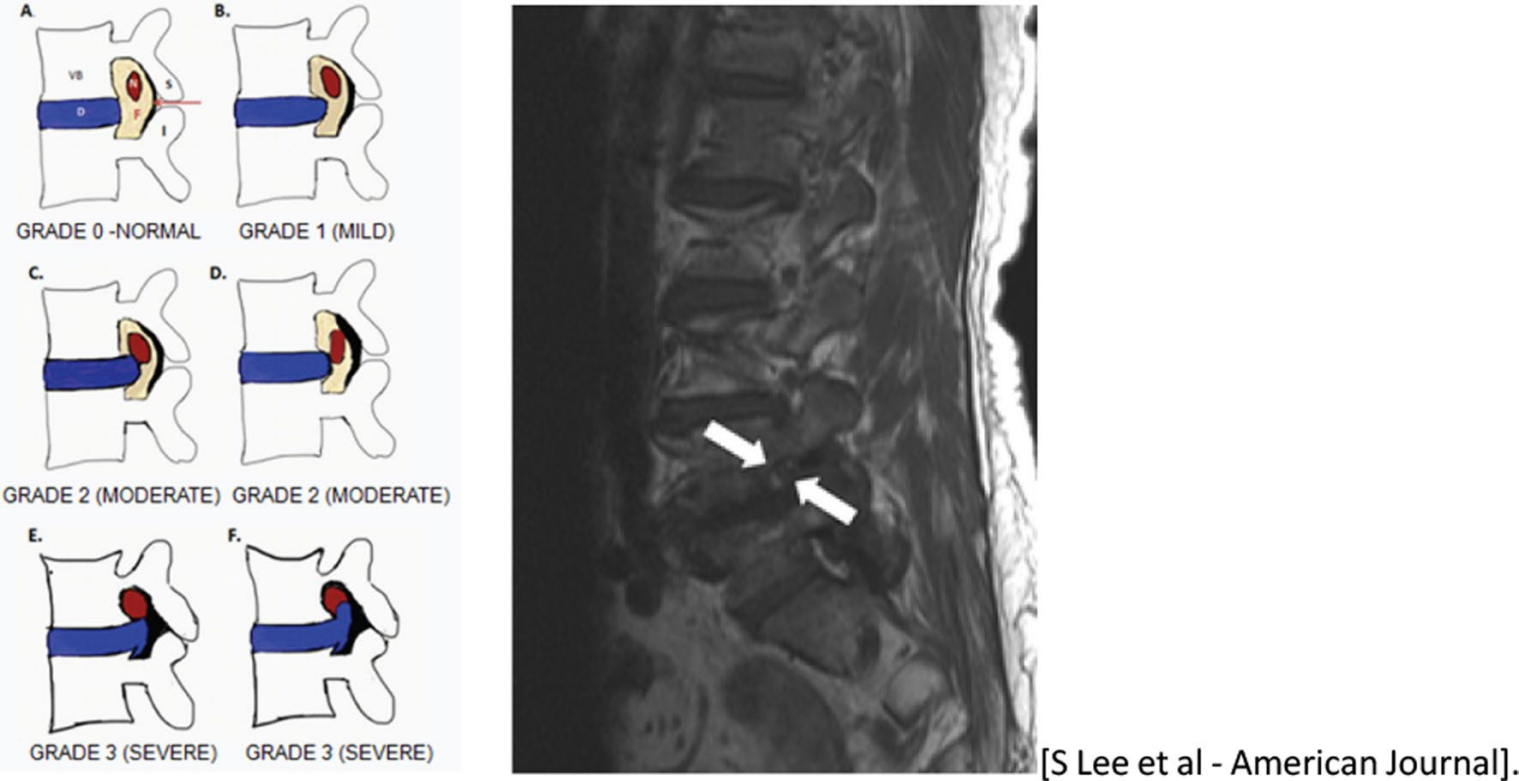
Schematic diagram showing the peri-neural fat surrounding the nerve root in grade zero stenosis. Grade one (mild) stenosis is showing partial loss of the peri-neural fat whereas grade two (moderate) and grade three (severe) stenosis are showing circumferential loss of peri-neural fat. The T1W sagittal MRI image is showing a partial loss of the epidural fat surrounding the nerve root in the left neuroforamen at L4/L5

**Fig. 6:**
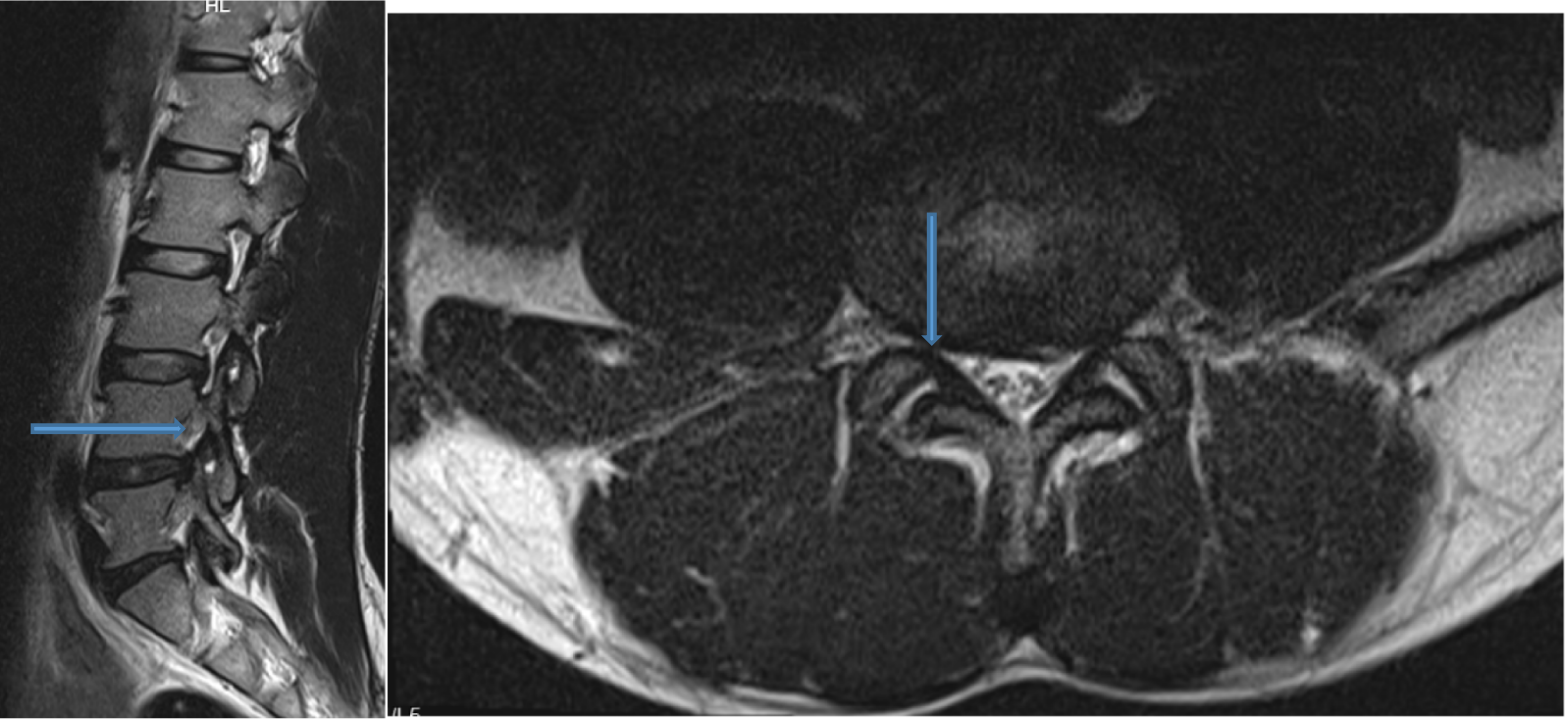
Sagittal and axial planes of T2W showing bilateral thinning out of the nerve roots with associated partial loss of epidural fat in the neuroforamina at L4/L5 (blue arrows)

**Fig. 7:**
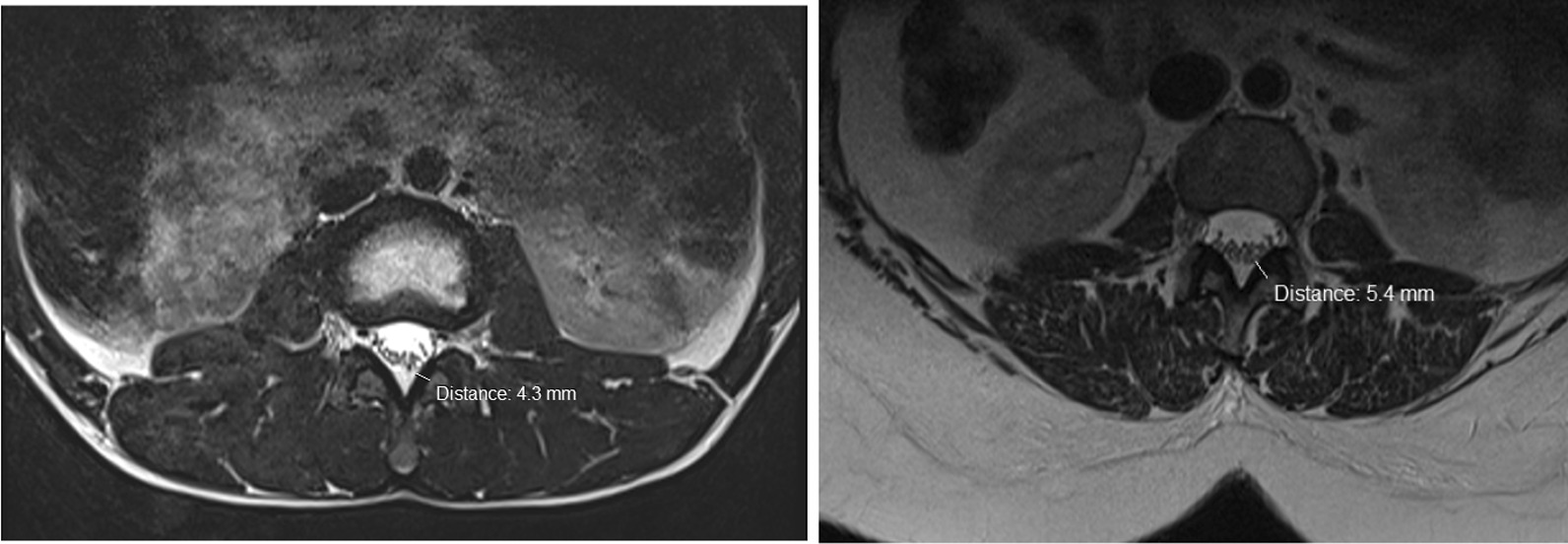
Axial plane of T2W showing ligamentum flavum hypertrophy at L3

**Fig. 8:**
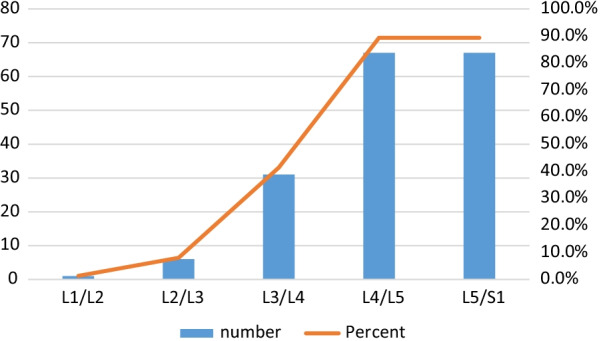
Facet joint effusion increased in frequency in the lower lumbar spine segment

**Fig. 9:**
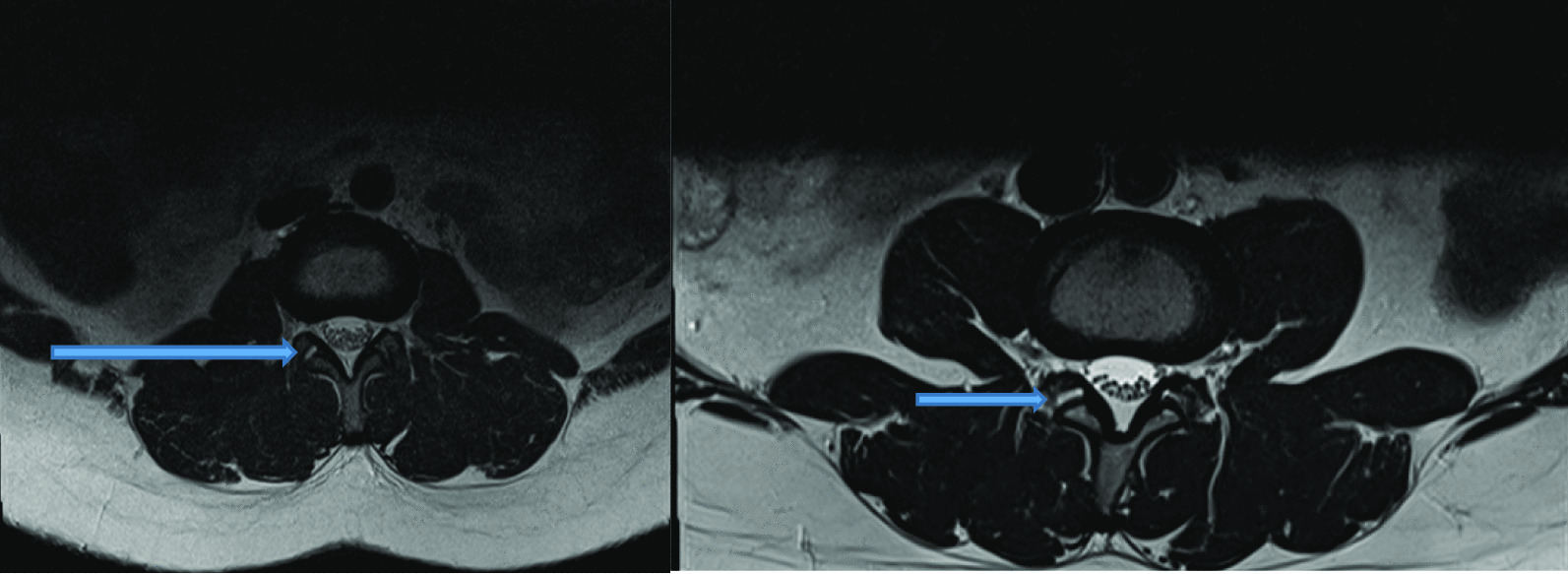
Axial plane of T2W showing bilateral facet joint effusion at L2/L3

Intervertebral disc (IVD) protrusion was observed in 48 (1.9%) patients at levels L3/L4, L4/L5 and L5/S1 [as shown by Fig. 2]. Only five patients had extrusion at L2/L3 IVD. Three (1.9%) had IVD migration at levels L4/L5 (n = 2) and L5/S1 (n = 1). Schmorl’s node was observed in 16 (10.2%) patients [as shown by Fig. 3]. There was no spondylolisthesis observed in all patients. Two (1.3%) patients had annular fissure at L1/L2 (n = 1) and L2/L3 (n = 1). Twenty (12.7%) patients had Modic degeneration; Type one (n = 13) and type two (n = 7) changes where both common at L4 and L5. Six patients had reduced vertebral heights. Five (3.2%) patients had vertebral body destruction at the level of L5 (n = 1), L4 (n = 2), L3 (n = 1) and L1 (n = 1) [as shown by Table 3 and Fig. 4]. Spinal canal narrowing of (< 10 mm) was observed in1 (4.5%) patient. Conus medullaris was at the level of L1/L2 in a majority (n = 147, 93.6%) of the patients. 39 (25%) patients had nerve root compression [as shown by Figs. 5 and 6]. Paravertebral area was affected in only 3 (1.9%) patients. There was no posterior element abnormality observed in all patients. Loss of normal lumbar lordosis was observed in 102 (65%) patients. Twenty (12.7%) patients had ligamentum flavum hypertrophy [as shown by Fig. 7]. Seventy-five (47.8%) patients had facet joint arthropathy at the following levels; L1/L2 (n = 1, 1.3%), L2/L3 (n = 6, 8%), L3/L4 (n = 31, 41.3%), L4/L5 (n = 67, 89.3%), and L5/S1 (n = 67, 89.3%) [as shown by Figs. 8 and 9].

**Table 3:** MRI findings of the patients

Variable	Frequency	Percentage
*Normal lumbar vertebral alignment*	N = 157	
Yes	142	90.4
No	15	9.6
*Intervertebral disc desiccation*	N = 157	
Yes	65	41.4
No	92	58.6
*Level of intervertebral disc desiccation*	N = 65	
T12/L1	2	3.1
L1/L2	6	9.2
L2/L3	11	16.9
L3/L4	17	26.2
L4/L5	43	66.2
L5/S1	49	75.4
*Intervertebral disc bulge*	N = 157	
Yes	49	31.2
No	108	68.8
*Level of intervertebral disc bulge*	N = 49	
L1/L2	34	69.2
L2/L3	36	73.1
L3/L4	40	81.3
L4/L5	48	98.7
L5/S1	49	100
*Severity of disc bulge*	N = 49	
L1/L2		
Mild	48	98.7
Moderate	1	0.7
Severe	1	0.7
L2/L3		
Mild	47	96.1
Moderate	1	2.6
Severe	1	1.3
L3/L4		
Mild	34	70.6
Moderate	13	26.1
Severe	02	3.3
L4/L5		
Mild	10	20.3
Moderate	20	41.2
Severe	18	38.6
L5/S1		
Mild	08	16.3
Moderate	18	37.3
Severe	23	46.4
*Schmorl’s node*	N = 157	
Yes	16	10.2
No	141	89.8
*Schmorl’s node position*	N = 16	
Anterior	8	50
T12	1	12.5
L1	2	25.0
L2	1	12.5
L5	4	50.0
Posterior	8	50
L2	2	12.5
L3	3	37.5
L5	3	37.5
*Spinal canal narrowing*	N = 15	
Mild	12	81.5
Moderate	02	14.0
Severe	01	4.5
*Nerve root compression*	N = 157	
Yes	39	25
No	118	75
*Nerve root compression*	N = 39	
Bilateral	30	76.1
Unilateral	09	23.9
*Marginal osteophytes*	N = 157	
Yes	50	31.8
No	107	68.2
*Levels of marginal osteophytes*	N = 50	
L1	21	42.0
L2	34	68.0
L3	42	84.0
L4	40	80.0
L5	40	80.0

**Fig. 10:**
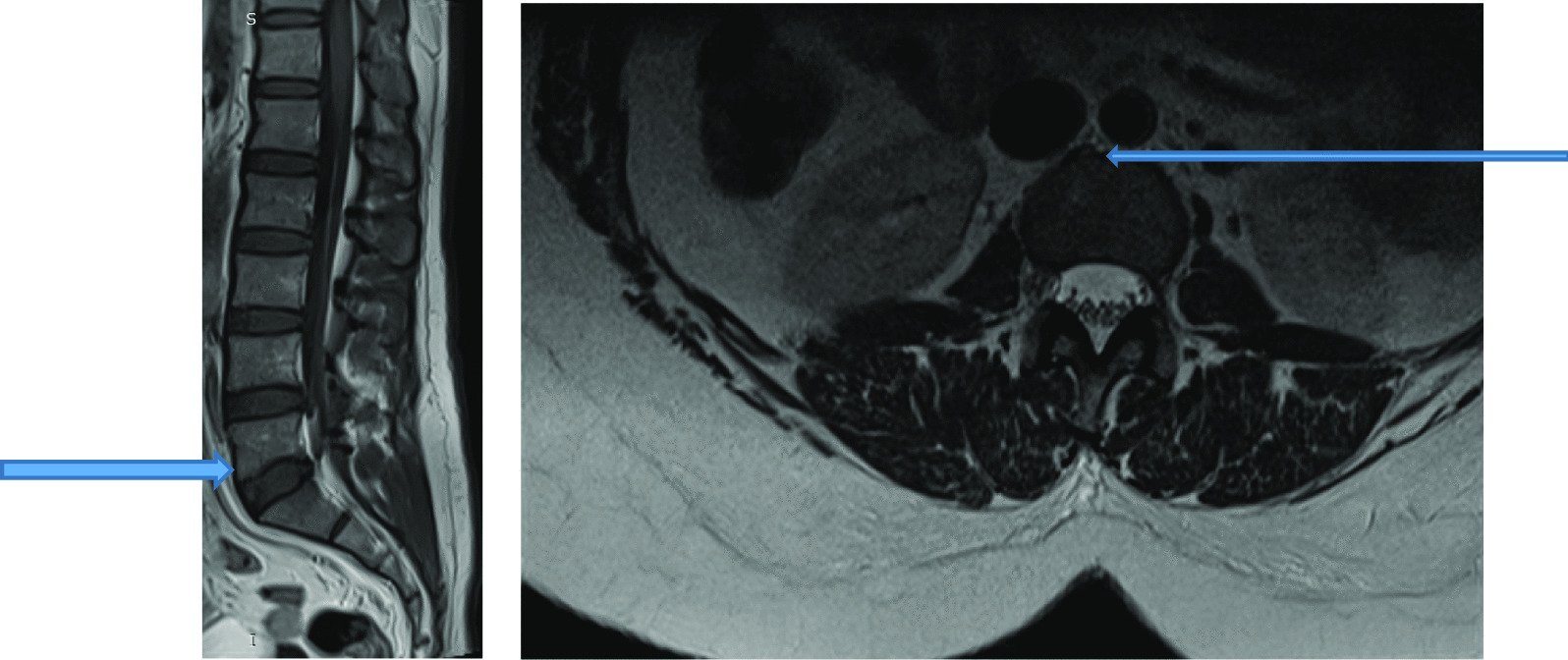
T1W and T2W in sagittal plane; and axial plane of T1W showing new bone formation [osteophytosis] (blue arrows) at the margins of L3, L4 and L5 in a 22 years old female patient who presented with low back pain for 1 month

**Fig. 11:**
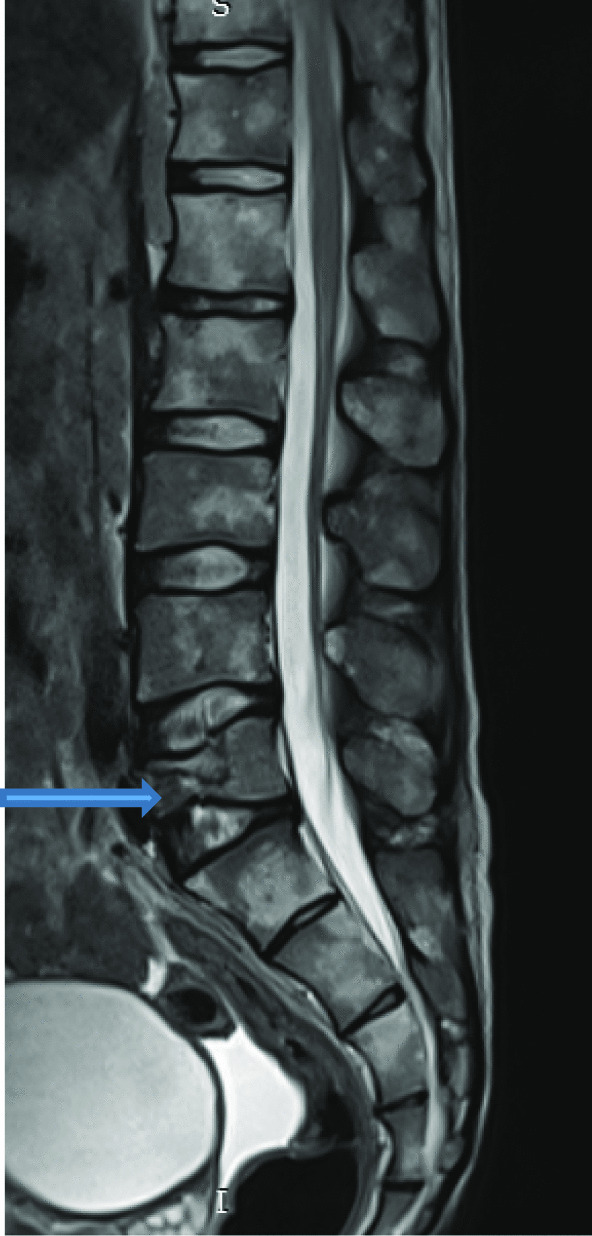
Sagittal plane of lumbar spine showing compressed fracture of anterior column of L5 vertebra (blue arrow)

Seventy-five (56.3%) patients had multiple level disease, commonly involving L4/5 and L5/S1 levels [as shown by Figs. 10 and 11].

**Fig. 12:**
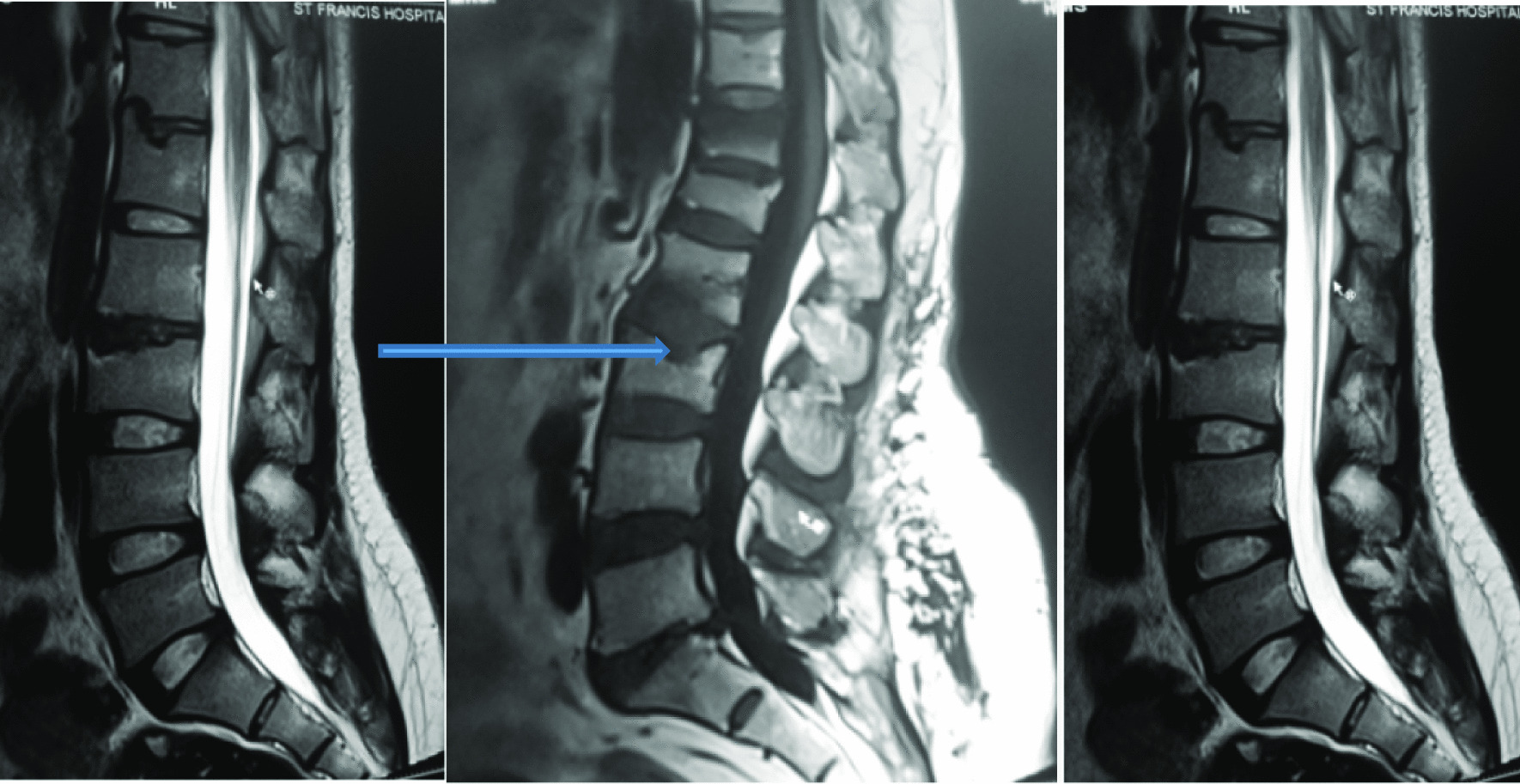
T2W sequence in Sagittal plane showing disc desiccation at L2/L3 and Schmorls node (blue arrow) at vertebrae L1 and L3

**Fig. 13:**
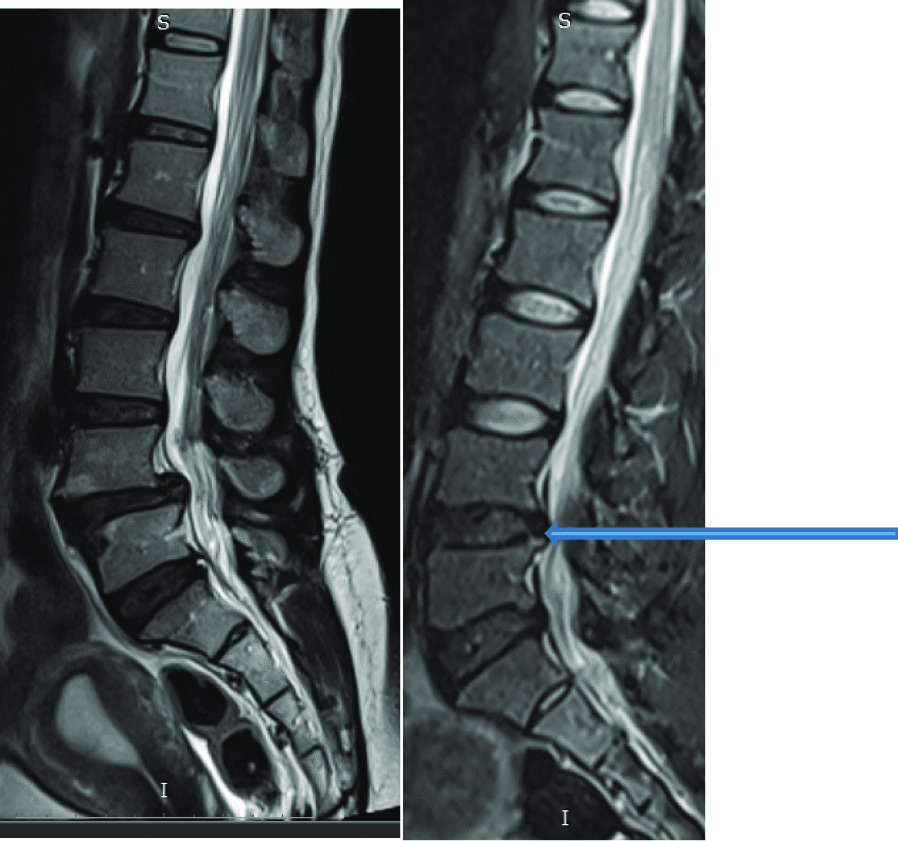
Sagittal plane of T2W sequence showing misalignment with disruption of both anterior and posterior vertebral lines at L4/L5 and L5/S1; and disc desiccation at L1/L2, L2/L3, L3/L4, L4/L5 and L5/S1 in a 34 years old male patient who presented with low back pain for 2 months

The most common MRI feature observed was reduced disc signal intensity [as shown by Figs. 12 and 13]. Overall, the L4/L5 disc, was diseased in the majority of the patients (80%) and was more frequently seen in the higher (25 to 39 years) age group (see Table 3).

### Correlation between MRI finding and clinical characteristics

Under bivariate analysis, patients’ age and pain distribution were associated with disc desiccation.

The age group of 23–35 years was associated with presence of disc desiccation. The risk of patients developing disc desiccation was found to increase with age. Patients in the age group of 23–35 years were 1.81 times more likely to develop disc desiccation than those in age group of 18–22 years and the difference between the two age groups was statistically significantly (*p* = 0.002). Those in the age group of 35–40 years were 2.22 times more likely to develop disc desiccation when compared to other age groups and the difference was statistically significant (*p* = 0.022).

Distribution of pain also predicted the occurrence of disc desiccation under bivariate analysis in this study. Those who were presenting with radiating pain were 2.58 times more likely to have disc desiccation than those with localized low back pain (49 cases versus 17 cases) and the difference was statistically significant (*p* = 0.006) as shown in Table 4.

**Table 4:** Logistic regression under bivariate analysis for the association of the independent factors with disc desiccation in the patients

Variables	Disc desiccation		95% CI	COR	*p*
	Yes	No			
*Age (years)*					
18–24	2	17	1	1	
25–34	29	48	2.427–53.940	1.81	0.002
35–39	35	26	1.123–4.423	2.22	0.022
*Sex*					
Male	30	47	0.413–1.473	0.78	0.443
Female	36	44			
*Occupation*					
Formal	37	47	0.721–2.576	1.36	0.34
Informal	29	44			
*Onset*					
Gradual	40	47	0.758–2.738	1.44	0.265
Sudden	26	44			
*Type of pain*					
Acute	49	76	0.260–1.243	0.57	0.54
Chronic	17	15			
*Duration of pain*					
≤ 10	59	86	0.148–1.618	0.49	0.234
> 10	7	5			
*Site of pain*					
Unilateral	24	28	0.658–2.514	1.286	0.462
Bilateral	42	63			
*Quality of pain*					
Aching	15	26	0.353–1.531	0.74	0.411
Burning	51	65			
*Severity of pain*					
Moderate	11	14	0.464–2.605	1.10	0.828
Severe	55	71			
*Limb weakness*					
Yes	4	2	0.510–16.163	0.51	0.213
No	62	89			
*Sensory loss*					
Yes	1	0	0.207–2.922	0.24	0.2315
No	64	91			
*Pain distribution*					
Radiating	49	48	1.297–5.139	2.58	**0.006**
Localized	17	43			

When the two independent factors (age and distribution of pain) were subjected to multivariate analysis and after controlling for all other independent factors, it was found that, both factors remained predictors of the outcome (disc desiccation). The risk of the patients with age group 25–34 years was 11.42 times more than those in the age group of 18–24 years and the difference was statistically significant (*p* = 0.004). Likewise, the odds of the patients in the age group of 35–40 years to develop disc desiccation was 2.42 more than those in the age group of 18–24 years and the difference was statistically significant (*p* = 0.029). Distribution of pain among the patients continued to be associated with disc desiccation in the study. The risk of patients with radiating low back pain was 1.23 times more than those with localized low back pain with statistical significance difference (*p* = 0.024) as shown in Table 5.

**Table 5:** Logistic regression under multivariate analysis for the association of the independent and the dependent variables

Variables	Disc desiccation		95% CI	AOR	*p*
	Yes	No			
*Age (years)*					
18–24	2	17	1	1	
25–34	29	48	2.159–60.447	11.42	0.004
35–39	35	26	1.097–5.347	2.42	0.029
*Pain distribution*					
Radiating	49	48	0.169–0.885	1.23	0.024
Localized	17	43			

None of the independent factors that were associated with presence of disc bulging in the patients for both bivariate and multivariate analyses were statistically significant as shown in Table 6. However, two of the variables analyzed permitted multivariate analysis to be performed due to the reason that, they had *p* values which were not exceeding 0.2 in bivariate analysis.

**Table 6:** Bivariate analysis using logistic regression for the association of the independent factors with disc bulging in the patients

Variables	Disc bulging		95% CI	COR	*p*
	Yes	No			
*Age (years)*					
18–24	5	15	1	1	
25–34	21	56	0.203–16.113	0.701	0.584
35–39	24	37	0.413–3.273	0.641	0.665
*Sex*					
Male	21	56	0.353–1.375	0.696	0.297
Female	28	52			
*Occupation*					
Informal	27	54	0.623–2.417	0.449	0.554
Formal	22	54			
*Onset*					
Gradual	30	57	0.999–1.072	1.029	0.297
Sudden	19	51			
*Type of pain*					
Acute	37	88	0.311–1.579	0.701	0.391
Chronic	12	20			
*Duration of pain*					
≤ 10 weeks	44	101	0.184–2.077	0.610	0.420
> 10 weeks	5	7			
*Site of pain*					
Unilateral	28	34	0.622–2.567	0.341	0.517
Bilateral	21	74			
*Quality of pain*					
Aching	17	24	0.885–3.908	0.349	0.101
Burning	32	84			
*Severity of pain*					
Moderate	11	14	0.810–4.662	0.339	0.137
Severe	38	94			
*Limb weakness*					
Yes	3	3	0.444–11.737	0.438	0.323
No	46	105			
*Sensory loss*					
Yes	1	1	0.261–2.901	0.331	0.314
No	48	107			
*Pain distribution*					
Yes	29	68	0.428–1.702	0.853	0.652
No	20	40			

When multivariate analysis was done, none of the two variables, quality of pain and severity of pain were found to be statistically significant as shown in Table 7.

**Table 7:** Multivariate analysis for the association of the independent and dependent variables using logistic regression

Variables	Disc bulging		95% CI	AOR	*p*
	Yes	No			
*Quality of pain*					
Aching	17	24	0.815–5.079	0.329	0.128
Burning	32	84			
*Severity of pain*					
Moderate	11	14	0.845–5.380	O.319	0.109
Severe	38	94			

Marginal osteophytes were the other MRI findings examined in this study and their presence were associated with the different independent factors included in this study. Only duration of LBP was the predictor of marginal osteophytes under bivariate analysis. Patients who had low back pain for the duration more than 10 weeks were 99.93% more likely to be diagnosed with marginal osteophytes than those who had low back pain for ≤ 10 weeks and the difference was statistically significant (*p* = 0.006). Occupational and limb weakness both showed marginal association with marginal osteophytes (*p* = 0.06). There were more patients with informal occupation (37 patients) who had marginal osteophytes compare to 35 patients with formal occupation who had marginal osteophytes, however, the difference was not statistically significant (*p* = 0.06) and they were 20% more likely to have marginal osteophytes as shown in Table 8.

**Table 8:** Bivariate analysis using logistic regression for the association of the independent factors with marginal osteophytes in the patients

Variables	Marginal osteophytes		95% CI	COR	*p*
	Yes	No			
*Age (years)*					
18–24	6	13	1		
25–34	38	39	0.618–5.471	0.35	0.27
35–39	28	33	0.444–1.707	0.53	0.68
*Sex*					
Male	35	42	0.517–1.815	0.97	0.92
Female	37	43			
*Occupation*					
Formal	35	46	0.428–1.504	0.8	0.49
Informal	37	39			
*Onset*					
Gradual	34	53	0.286–1.022	0.54	0.06
Sudden	38	32			
*Type of pain*					
Acute	54	71	0.270–1.294	0.59	0.19
Chronic	18	14			
*Duration of pain*					
≤ 10 weeks	61	84	0.008–0.525	0.07	**0.001**
> 10 weeks	11	1			
*Site of pain*					
Unilateral	26	26	0.659–2.498	1.29	0.46
Bilateral	46	59			
*Quality of pain*					
Aching	21	20	0.655–2.732	1.34	0.42
Burning	51	65			
*Severity of pain*					
Moderate	14	11	0.868–3.842	1.62	0.27
Severe	58	74			
*Limb weakness*					
Yes	5	1	0.715–54.952	6.27	0.06
No	57	84			
*Sensory loss*					
Yes	0	1	1.613–2.162	1.87	0.35
No	72	83			
*Pain distribution*					
Yes	48	49	0.766–2.821	1.47	0.25
No	24	36			

After adjusting for other covariates in multivariate analysis, duration of pain became not predictive of marginal osteophytes (*p* = 0.96) although there were more patients with marginal osteophytes (61 cases) among those who had low back pain ≤ 10 weeks compared to the ones who had low back pain for the duration of > 10 weeks as shown below in Table 9.

**Table 9:** Multivariate analysis for the association of the independent and dependent variables using logistic regression

Variables	Marginal osteophytes		95% CI	AOR	*p*
	Yes	No			
*Onset of pain*					
Gradual	49	48	0.548–2.782	0.45	0.61
Sudden	17	43			
*Type of pain*					
Acute	54	71	0.368–3.254	0.48	0.87
Chronic	18	14			
*Duration of pain*					
≤ 10 weeks	61	81	0.219–4.921	0.49	0.96
> 10 weeks	11	1			
*Limb weakness*					
Yes	5	1	0.323–1.535	0.707	0.39
No	57	84			

## Discussion

We have reported on young adult patients with LBP in Nsambya hospital. The present study described the MRI findings in the lumbar spine which included decreased signal intensity, changes in disc contour (bulge, protrusion, extrusion and sequestration) marginal osteophytosis and facet joint osteoarthritis.

Disc desiccation was the most common disc finding in our study, and increased infrequency with age [5, 8] which explains the age-related higher frequency, when compared to other studies of young adults, especially those in (25–39 years) age group. Our observation is consistent with observations made in other studies conducted in other regions of the world on similar age groups [13]. Most patients in this study had multiple level disease with the highest prevalence (46%) at the 2 lowest lumbar levels (L4/L5 and L5/S1), which is consistent with observations made by others [8, 10, 13].

In a study from Finland on a group aged 20–22 years, Takatalo et al. [8] found a 47% prevalence of degeneration diseases of lumbar spine. Savage et al. [24] from UK compared MRI features between 2 age groups (20–30 years versus 31–59 years) in the 20 to 30-years-age group, they found that 34% of subjects had disc diseases on MRI compared to 59% in the older age group. A study on Chinese juveniles (13–20 years) reported 35% of spinal disc degeneration [25]. Osama Al-Saeed et al. [13] from Kuwait also compared MRI features between the cases and control groups (16–29 years), they found that 64% had disc degeneration.

In the current study, the frequency of MRI lumbar spine disc changes appears to be higher (47.7%) when compared to other reports in literature [13, 25, 26] and these changes were more frequent (62%) in the higher (25–39 years) age group. This can partly be due to their involvement in intense activities, and lack of physical loading that would cause these changes and relate to the back problems [27, 28]. There are reports that shows that low physical activity (being sedentary) is also associated with increased occurrence of disc degeneration [29] and higher prevalence of LBP in the young adult population [30]. There are also studies which reports that high activities such as competitive sports correlates with increased occurrence of MRI findings of disc abnormality in young adults [31]. Heavy works have also been found to be a risk factor for disc degeneration [12]. In the present study, 79% of the patients reported to be doing moderate to severe (intense) activities, and majority of whom were men. There are studies which reports that high environmental temperatures especially in the tropics is associated with development of disc desiccation [32].

The non-disc changes of degeneration that was observed comprised marginal osteophytes, foraminal stenosis with nerve root compression, end plate irregularities, spinal canal stenosis and facet joints arthropathy. Facet joint effusion (48%) and osteophytes (32%) were the most common. Our observation is consistent with observations from other studies [13]. Literature shows that non-disc changes of degeneration disease increases in frequency with age [14, 33]. These changes are attributed to heavy works and intense activities [15].

Statistical analysis confirms that age, pain distribution and duration of pain as the independent factors which associated with abnormal MRI findings in our study. This is supported by a large population based study from Norway [32], Middle East [13] and China [25] which revealed a high prevalence of MRI findings associating with history of chronic pain in the lumber-sacral region. Literature shows that chronic clinical features of LBP is associated with most features on MRI. This fact is also supported by the current study where patients with history of chronic back pain where found to have MRI features of disc degeneration disease.

### Study limitations

1. The sample size was small to give a general picture of Uganda’s population.
2. Several images with MR findings that would aid in the understanding of the findings were absent.

## Conclusion

Pain lasting more than 10 weeks is the most common clinical presentation among patients with LBP.

Disc contour irregularity, disc desiccation and loss of disc height are the most common findings in patients who present with severe LBP of acute onset in this study.

Disc bulge is the most common MRI finding in patients with LBP.

Disc desiccation is common among patients above 24 years of age who present with LBP. However, there is no association between sex, occupation and activity levels with disc desiccation.

Disc bulge and marginal osteophytes show no association with age, occupation and activity levels.

LBP which is radiating in nature is the most common finding in patients with disc protrusion in this study.

Severe burning pain of gradual onset is common among patients with severe disc bulge and osteophytes.

## Data Availability

Raw data and materials are available on request of the corresponding author.
